# Controlling response order without relying on stimulus order – evidence for flexible representations of task order

**DOI:** 10.1007/s00426-024-01953-w

**Published:** 2024-04-13

**Authors:** Jens Kürten, Tilo Strobach, Lynn Huestegge

**Affiliations:** 1 Department of Psychology, University of Würzburg, Roentgenring 11, 97070 Würzburg, Germany; 2 Department of Psychology, MSH Medical School Hamburg, Am Kaiserkai 1, 20457 Hamburg, Germany

## Abstract

In dual-task situations, both component tasks are typically not executed simultaneously but rather one after another. Task order is usually determined based on bottom-up information provided by stimulus presentation order, but also affected by top-down factors such as instructions and/or differentially dominant component tasks (e.g., oculomotor task prioritization). Recent research demonstrated that in the context of a randomly switching stimulus order, task order representations can be integrated with specific component task information rather than being coded in a purely abstract fashion (i.e., by containing only generic order information). This conclusion was derived from observing consistently smaller task-order switch costs for a preferred (e.g., oculomotor-manual) versus a non-preferred (e.g., manual-oculomotor) task order (i.e., order-switch cost asymmetries). Since such a representational format might have been especially promoted by the sequential stimulus presentation employed, we investigated task-order representations in situations without any bottom-up influence of stimulus order. To this end, we presented task stimuli simultaneously and cued the required task-order in advance. Experiment 1 employed abstract order transition cues that only indicated a task-order repetition (vs. switch) relative to the previous trial, while Experiment 2 used explicit cues that unambiguously indicated the task-order. Experiment 1 revealed significant task-order switch costs *only* for the second task (of either task order) and no order-switch cost asymmetries, indicating a rather generic representation of task order. Experiment 2 revealed task-order switch costs in *both* component tasks with a trend toward order-switch cost asymmetries, indicating an integration of task order representations with component task information. These findings highlight an astonishing flexibility of mental task-order representations during task-order control.

When performing two tasks in close temporal proximity, commonly referred to as dual tasking, we are often slower and/or more error-prone than when executing a single task in isolation (Pashler, 1994). This is particularly troublesome in modern society, where the ability to multitask needs to be maintained throughout both work and leisure time (Schumann et al., 2022). However, apart from keeping such performance decrements to a minimum, there is another fundamental prerequisite to deal with multitasking requirements: We seldom execute the actions of both tasks at the exact same time, but rather one after another, indicating a need to schedule tasks (i.e., task-order control). In typical dual-task studies, participants rely on the presentation order of sequential task stimuli to schedule tasks. It is thus a viable strategy to integrate features of component tasks (e.g., relevant effector systems) within representations of task order. The present study addresses how task order is represented in situations which do not promote the use of stimulus sequence to determine task order, that is, in which task order rather needs to be controlled in purely a top-down manner.

## Task-order control in the PRP paradigm

Dual-task performance has been traditionally studied in the psychological refractory period paradigm (PRP paradigm; Telford, 1931) which is characterized by the sequential presentation of two task stimuli (S1 and S2) that indicate each component task’s response (R1 and R2). The hallmark PRP finding is a decreasing dual-task performance cost for Task 2 as the time between the two stimuli (the stimulus onset asynchrony, SOA) increases. This so-called PRP effect has been interpreted in terms of processing limitations due to a structural (e.g., Pashler, 1994; Pashler & Johnston, 1989) or strategic (Logan & Gordon, 2001; Meyer & Kieras, 1997) *bottleneck* only allowing for one response selection at a time (e.g., Pashler, 1994), or in terms of the sharing of limited *mental capacity* and the resulting decision to perform tasks sequentially (e.g., Logan & Gordon, 2001; Meyer & Kieras, 1997; Navon & Miller, 2002; Tombu & Jolicœur, 2003). However, regardless of whether sequential task execution is the result of a structural limitation or a strategy, any task sequence needs to be scheduled, requiring some sort of task-order control, an issue often neglected in PRP studies. Early bottleneck models (Pashler, 1994) assumed that task execution order was determined in a mainly *bottom-up* manner by copying the (usually constant) stimulus order, sometimes referred to as the first-come-first-served principle (Sigman & Dehaene, 2006; Strobach et al., 2018). However, the influence of *top-down* factors on task order (i.e., active task-order control) became evident from studies comparing constant task (stimulus) order with varying task (stimulus) order (e.g., De Jong, 1995; Luria & Meiran, 2003, 2006; Szameitat et al., 2006). For example, De Jong (1995) demonstrated a tendency to repeat the response order of the previous trial in mixed stimulus order blocks when stimulus presentation order switched. In addition, response-order reversals were shown to occur particularly often with certain combinations of effector systems. Pieczykolan and Huestegge (2019) showed that oculomotor responses were frequently executed prior to manual responses, even when the stimulus for the oculomotor task came second (i.e., response-order reversals), indicating a clear top-down preference for some response orders over others (e.g., oculomotor-manual > manual-oculomotor). The tendency toward response-order reversals in these studies was reduced by giving explicit instructions to respond in accord with stimulus order versus a free order choice instruction, again highlighting the strategic element of top-down task-order control (see also Kübler et al., 2018). Indeed, typical PRP studies employ such an explicit order instruction (e.g., Hommel, 1998; Pashler & Johnston, 1989), thereby conflating bottom-up factors (i.e., stimulus order) and top-down factors (i.e., strategic adjustments) of task order control. All this previous research indicates more active, top-down task-order control processes (in addition to a purely bottom-up “first-come, first-served” principle) that is often assumed to occupy a separate processing stage (“task-order control stage”) before the processing of individual component tasks (De Jong, 1995; Luria & Meiran, 2003, 2006; Sigman & Dehaene, 2006). However, within a typical PRP-style setting, any influence of bottom-up task-order control cannot be eliminated completely as stimuli are presented sequentially (for a reduction of bottom-up influences on task-order control, see Luria & Meiran, 2006, Experiment 2). Thus, for a more complete picture of task-order control processes it seems necessary to investigate how task order is controlled in a setting without the requirement to rely on stimulus order information.

## Task-order representations – separate from or integrated with component tasks

Beside the tendency to reverse response order, situations with randomly switching stimulus order (paired with the instruction to respond in accord with stimulus order) can incur substantial costs in other performance parameters. For example, performance in both the first and the second component task was hampered in task-order repetitions (i.e., trials in which the task order corresponds to that in the previous trial) in blocks with randomly changing task order (i.e., mixed-order blocks) relative to blocks with a constant task order (i.e., fixed-order blocks) indicating a general task-order coordination cost (Luria & Meiran, 2003). Such task order coordination costs are likely associated with the need to maintain readiness for and to coordinate two (or more) task orders in working memory (Los, 1996) and not with the process of reconfiguring task order per se (Luria & Meiran, 2003). Additionally, however, in mixed-order blocks, performance in both component tasks is usually worse in task-order switches (relative to the previous trial) compared with task-order repetitions (Huestegge et al., 2021; Kübler et al., 2018; Luria & Meiran, 2003, 2006; Szameitat et al., 2006). These task-order switch costs are typically taken as evidence for the costly reconfiguration of explicit *task-order representations* within a task-order *set* (Kübler et al., 2022a; Szameitat et al., 2006) and the fact that *both* component tasks are usually affected by an order switch supports the notion that this reconfiguration happens before the first component task is being processed (Luria & Meiran, 2003, 2006). Task-order coordination costs and task order switch costs are closely related to the notion of mixing costs and switch costs in the literature on single-task switching (see Kiesel et al., 2010; Vandierendonck et al., 2010 for reviews), and the idea of a mental task-order set containing representations of different task orders are reminiscent of respective parallels in the literature on single-task switching. However, it is important to note that if only single tasks were represented and these representations were reconfigured, one would not expect to find task-order switch costs in a PRP trial (at least for the first component task) as a task-order switch would result in a local component task repetition across trials (e.g., oculomotor-[manual ◊ manual]-oculomotor). Thus, task-*order* representations and effects of task-order switching are assumed to reside on a “higher level” than single task representations (Hirsch et al., 2017, 2018; Kübler et al., 2022b; Strobach, 2023; Strobach et al., 2021). The present study investigated whether such higher-level task-order representations contained information about the to-be-scheduled component tasks (“Task A – Task B” vs. “Task B – Task A”), or whether they contain only generic order information (“0–1” vs. “1 − 0”), and to what extent task-order representations can be flexible in situations requiring purely top-down task-order control.

Huestegge and colleagues (2021) studied the content of task-order representations in a classic PRP paradigm with randomly ordered, sequentially presented stimuli and the instruction to respond in accord with the stimulus order (see also Kübler et al., 2022b; Luria & Meiran, 2003, 2006; Strobach et al., 2018, 2021). Importantly, they employed two differentially dominant component tasks (oculomotor > manual), thus creating a clear preference for executing one task order over another (e.g., oculomotor-manual > manual-oculomotor). The main findings were significantly smaller task-order switch costs when switching to the preferred (oculomotor-manual) versus to the non-preferred (manual-oculomotor) task order in both component tasks. Such order-switch cost asymmetries indicated that task-order representations contained information about the specific component task characteristics and that task-order information and component task information are thus represented in an integrated fashion. Essentially, the oculomotor-manual task order was configured more easily (i.e., faster) than the manual-oculomotor task order, which is incompatible with the assumption of a purely generic task order representation (e.g., switching between a generic “0–1” code to a “1 − 0” code and vice versa should come at the same cost). Task-order coordination costs, on the other hand, were not affected by the specific task order being switched to, again suggesting that these costs are more a result of the need to maintain two (vs. only one) task-order representation in working memory. Taken together, tasks that can be distinguished based on separate, sequentially presented stimuli as well as differentially dominant component tasks seem to be most conducive to an integrated representation of task order. In contrast, comparable task-order switch costs for transitioning to either task order would indicate a more generic task-order representation, which might be observed in absence of a strong preference for executing one task order over another (see Kübler et al., 2022a for evidence for more generic task-order representations with highly similar tasks). Importantly, presenting task stimuli sequentially as is typical for studies on task-order control might particularly promote an integrated task-order representation due to the clear separation of component task information. It is thus crucial to determine whether task order might be represented differently, if no bottom-up information about task-order is present, that is, if task order needs to be controlled in a purely top-down fashion.

## Present study

Based on the considerations above, the present study was conducted to address two main issues. First, we wanted to investigate the effects of top-down task-order control requirements on performance in a setting without the opportunity for using bottom-up information of stimulus order. Second, and more critically, we wanted to determine the format of task-order representations (i.e., integrated with or separate from component tasks) in such a situation. To this end, participants responded to one of two simultaneously presented visual stimuli with an eye-movement in the (dominant) oculomotor task and to the other with a key press in the (non-dominant) manual task, thereby creating two differentially preferred task orders (oculomotor-manual > manual-oculomotor). Task order was signaled to the participant by means of a cue prior to stimulus onset. In Experiment 1, we used highly abstract order transition cues that only signaled the requirement to repeat or switch task order relative to the previous trial, thereby promoting a generic task-order representation. In Experiment 2, we used explicit task-order cues that contained information about the component tasks to be scheduled. Note that previous studies on task-order control have also employed cues (e.g., De Jong, 1995; Luria & Meiran, 2003, 2006; Steinhauser et al., 2021); however, in these studies the cue always indicated the (likely) first stimulus while response order was instructed to be in accord with the order of two sequentially presented stimuli (i.e., allowing for the emergence of bottom-up task-order control). Furthermore, these studies have never used differentially preferred task orders, precluding the analysis of potential cost asymmetries.

The main predictions are as follows. A preference for the oculomotor-manual (vs. manual-oculomotor) task order (as demonstrated by previous studies) should be reflected in a lower frequency of response reversals in the former (vs. the latter) task-order condition. Task-order coordination costs in terms of worse performance in order-repetition trials in mixed-order blocks compared with performance in fixed-order blocks would indicate the presence of two distinct task-order representations in mixed-order blocks. Overall, we would expect task-order switch costs in mixed-order blocks in both component tasks, in line with previous research. However, regarding the content of task-order representations in the present setting, representations of task order that are integrated with component task features would be reflected in asymmetries in task-order switch costs for transitioning to a preferred versus a non-preferred task order (e.g., order-switch costs should be smaller for the preferred oculomotor-manual task order than for the non-preferred manual-oculomotor task order). Such an asymmetry should be in the same direction for *both* component tasks (oculomotor task and manual task) based on previous findings of integrated task-order representations (Huestegge et al., 2021). In contrast, generic task-order representations that do not contain any information about component-task features should rather lead to symmetric task-order switch costs in both the preferred and the non-preferred task order. Any differences in the result patterns between Experiment 1 (using abstract order-transition cues) and Experiment 2 (using explicit task-order cues) would further indicate that the way task order is represented is not necessarily fixed and tied to a certain paradigm but can be rather flexible even in comparable contexts.

## Experiment 1

Experiment 1 made use of highly abstract task-order transition cues that only signaled information about whether to repeat or switch task order relative to the previous trial, thereby promoting generic task-order representations.

### Methods

#### Transparency and openness

We report how we determined our sample size, all data exclusions (if any), all manipulations, and all measures in the study. Preregistration information, raw data, and analysis scripts can be obtained from: https://osf.io/mwu8r/.

#### Participants

Fourty volunteers (mean age = 26 years, SD = 5.6, 78% female, 95% right-handed) took part in exchange for monetary compensation after giving written informed consent. All had normal or corrected-to normal vision. In accord with preregistered criteria, data sets of 7 participants were excluded (5 for an overall error rate > 33%, 2 for missing data) and recollected with the help of new participants to ensure full counterbalancing.

#### Apparatus, stimuli and procedure

Stimuli were presented on a black background at a viewing distance of about 67 cm on a 21-inch CRT monitor with a spatial resolution of 1024 × 768 pixels and a temporal resolution of 100 Hz. Eye-movements of the right eye were recorded using an Eyelink 1000 desktop-mounted eye tracking system (SR-Research Missisauga, Ontaria, Canada) with a sampling rate of 1000 Hz. A chinrest with forehead support was used to minimize head movements, and the eye tracker was recalibrated before each block. Manual responses were recorded using the left and right arrow key on a standard German QWERTZ keyboard. Participants went through 18 blocks of 48 trials each. The first two blocks were single-task blocks (oculomotor task OR manual task, order counterbalanced) in order to familiarize the participant with the setup but later discarded from all analyses. The remaining 16 dual-task blocks (oculomotor task AND manual task) comprised 8 fixed-order blocks (oculomotor -manual OR manual- oculomotor) and 8 mixed-order blocks (oculomotor-manual AND manual-oculomotor). The order of these blocks was counterbalanced to allow for a valid analysis of task-order coordination costs (comparison between fixed-order blocks and order-repetition trials of mixed-order blocks). By presenting fixed-order blocks both before *and* after mixed-order blocks, we can rule out practice and other general effects as the main cause of any observed effects.

**Table 1:** Four different sequences of block order that were randomly assigned to the participants

Sequence 1	Sequence 2	Sequence 3	Sequence 4
1x single oculomotor	1x single manual	1x single oculomotor	1x single manual
1x single manual	1x single oculomotor	1x single manual	1x single oculomotor
2x fixed order dual (ocul. → manual)	2x fixed order dual (ocul. → manual)	2x fixed order dual (manual → ocul.)	2x fixed order dual (manual → ocul.)
2x fixed order dual (manual → ocul.)	2x fixed order dual (manual → ocul.)	2x fixed order dual (ocul. → manual)	2x fixed order dual (ocul. → manual)
8x mixed order dual	8x mixed order dual	8x mixed order dual	8x mixed order dual
2x fixed order dual (ocul. → manual)	2x fixed order dual (ocul. → manual)	2x fixed order dual (manual → ocul.)	2x fixed order dual (manual → ocul.)
2x fixed order dual (manual → ocul.)	2x fixed order dual (manual → ocul.)	2x fixed order dual (ocul. → manual)	2x fixed order dual (ocul. → manual)

*Note* Numbers prior to block type refer to the respective number of blocks of trials

**Fig. 1:**
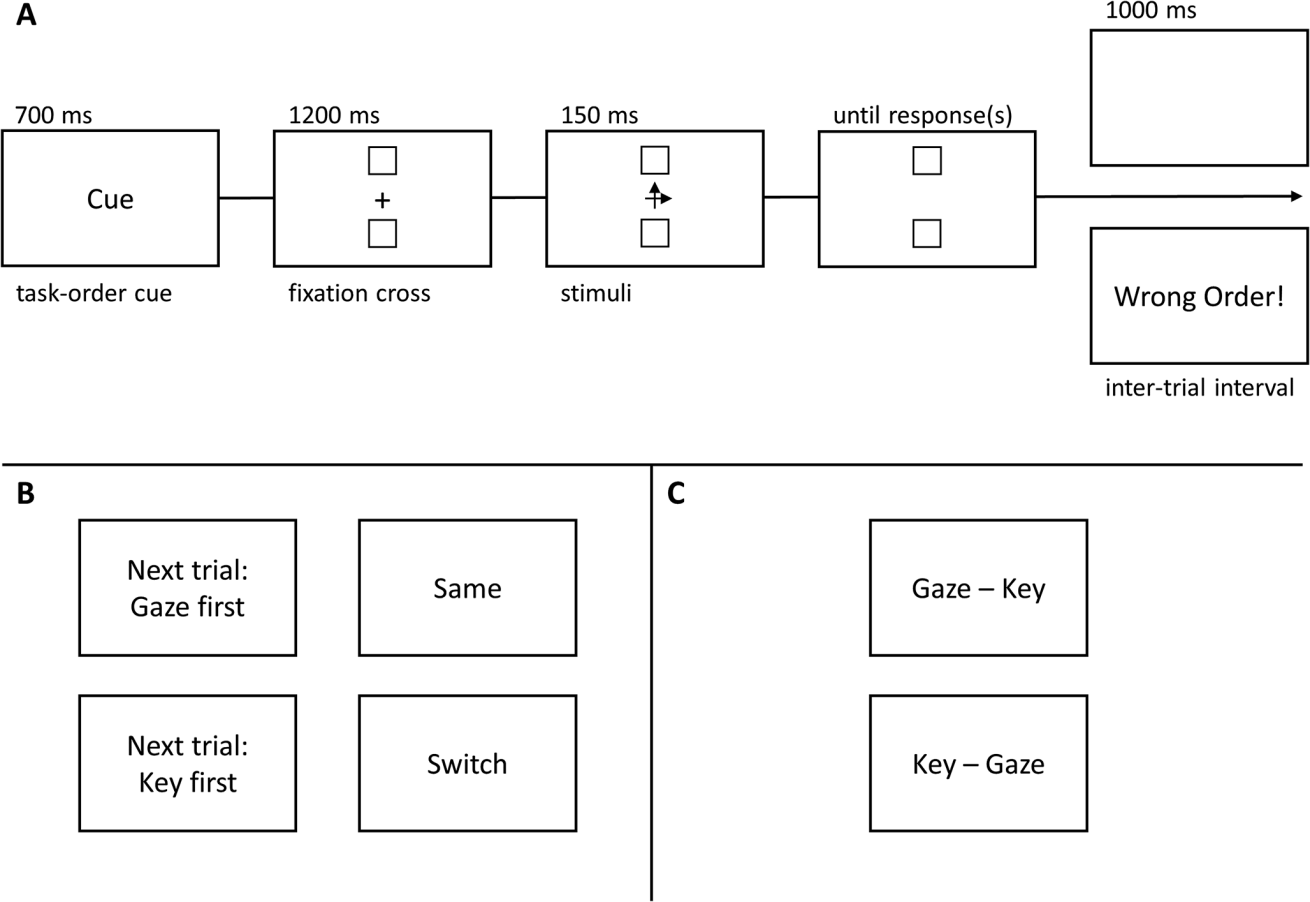
Schematic trial structure. *Note.* Time course of individual trials (Panel **A**). In Experiment 1 (Panel **B**), we used explicit task-order cues only in the first trial of each block and following a task-order reversal, and transition cues (“same” vs. “switch”) in all other trials. In Experiment 2 (Panel **C**), we used explicit task-order cues (“gaze-key” vs. “key-gaze”) throughout

Fig. 1A depicts the general trial structure of both experiments; cues used in Experiment 1 are presented in Fig. 1B. Each trial (regardless of block type) began with a task cue. The words “Nächster Durchgang: Taste zuerst” (German for “Next trial: Key first”), “Nächster Durchgang: Blick zuerst” (German for “Next trial: Gaze first”) were used as cues in the first trial of each block and in trials following an erroneous task-order reversal to explicitly indicate the required response order in the upcoming trial. The words “Wechsel” (German for “Switch”) and “Gleich” (German for ”Same”) were used as abstract cues in all other trials to indicate the requirement to switch or to repeat response order, respectively. In single-task blocks and fixed-order dual-task blocks, the “Same” cue was shown after every correct-order trial. The cue words (Times New Roman, font size 20) were presented in white for 700 ms. Following the cue presentation, a white plus sign (size = 10 px, 0.33° visual angle, VA) was presented in the center of the screen together with two white rectangles (size = 10 px, 0.33° VA) above and below at an eccentricity of 192 px (6.4° VA). After 1200 ms, the fixation cross was replaced by the simultaneous target stimuli consisting of two superimposed white arrow symbols (size = 0.4° VA each), one horizontal (→/←) and one vertical (↑/↓). The target stimuli were presented for 150 ms followed by a blank screen until both responses were issued. Participants placed the index finger and ring finger of their dominant hand on the left and right arrow keys and responded to the direction of the horizontal arrow with a spatially compatible key press. They responded to the direction of the vertical arrow with a saccade toward the spatially compatible white rectangle. The horizontal and vertical assignment to the manual and oculomotor tasks, respectively, is equivalent to the assignment in Huestegge et al. (2021, Experiment 2); we have chosen this assignment because it should increase the potential to find asymmetrical task-order switch costs (serving as evidence for integrated task-order representations). The instructions equally stressed speed and accuracy and, most importantly, a response order in accord with the cue. Trials with the correct response order were followed by a 1000 ms blank-screen inter-trial-interval (ITI), and the next trial’s cue. In case of a task-order reversal, the error message “Falsche Reihenfolge!” (German for “Wrong Order!”) was presented in red (Times New Roman font, size 20) at the screen center during the ITI, and the next cue explicitly indicated the required response order for the next trial.

#### Analytical details

To assess the general impact of task-order control on performance in the absence of any bottom-up influence of stimulus order, we analyzed task-order coordination costs in reaction times (RTs) and error rates (ERs) of both component tasks using 2 × 2 × 2 repeated measures ANOVAs with the within-subject factors task-order transition (fixed-order blocks vs. repetitions in mixed-order blocks), task order (oculomotor-manual vs. manual-oculomotor), and component task (oculomotor task vs. manual task). Note that response-order reversals (in %) can only be analyzed at the trial level and not at the component-task level because an order reversal in the oculomotor task is necessarily accompanied by an order reversal in the manual task and vice versa. Thus, response-order reversals were analyzed via a 2 × 2 repeated measures ANOVA with the within-subject factors task-order transition (fixed-order blocks vs. repetitions in mixed-order blocks) and task order (oculomotor-manual vs. manual-oculomotor). The main predictions of the current study, however, were related to the nature of task-order representations in situations requiring the frequent switch of task order (i.e., mixed-order blocks). To this end, task-order switch costs were analyzed in a further set of repeated measures ANOVAs with the within-subject factors task-order transition (repetition vs. switch) and task order (oculomotor-manual vs. manual-oculomotor) in response-order reversals, and – additionally – component task (oculomotor task vs. manual task) in RTs and ERs. These analyses were conducted in mixed-order blocks exclusively. All statistical analyses were performed at a significance level of α = 0.05. Significant interaction effects were analyzed further using simple effects analyses and Bonferroni-corrected pairwise comparisons. In cases of non-significant results regarding the critical task-order switch cost asymmetries (i.e., interaction effects of task-order transition and task order), we additionally computed Bayes Factors (BF01) using the BayesFactor package with a Cauchy scale parameter of 1.

### Results

**Fig. 2:**
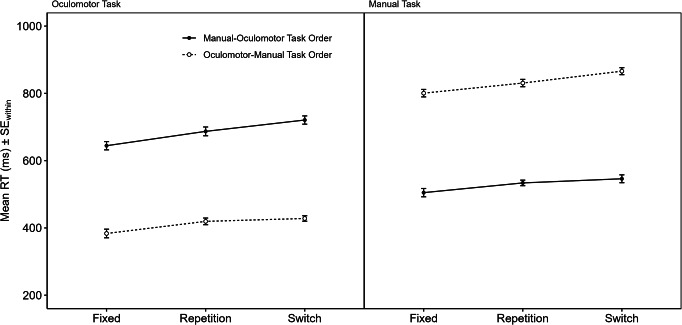
Mean RTs of Experiment 1 as a function of Task-Order Transition (Fixed vs. Repetition vs. Switch), Task Order (Manual-Oculomotor vs. Oculomotor-Manual), and Task (Oculomotor vs. Manual). *Note* For task-order switch cost asymmetries indicative of integrated task-order representations, the differences between Switch and Repetition trials would need to be greater for the Manual-Oculomotor Task Order (solid black lines) than for the Oculomotor Manual Task Order (broken lines) in *both* Tasks. Error bars represent within-subjects SEs following Cousineau-Morey corrections (O’Brien & Cousineau, 2014)

#### Data treatment

Oculomotor responses with an amplitude of > 1/3 of the distance toward the upper or lower rectangle (64 px, 2.14° VA) were counted as valid and were either classified as correct responses or directional errors. Oculomotor responses that fell short of that threshold were counted as omission errors (3.20% of all trials). The first trial of each block was excluded from all analyses since task order was always explicitly cued (2.08% of all trials). For the error and response reversal analyses, all remaining trials were considered. From RT analyses, trials containing response errors or response reversals and the immediately following trials were excluded (24.20% of all trials). Furthermore, trials with RTs < 150 ms and RTs > 3000 ms were excluded (1.37% of all trials). In total, 26.11% of all trials were excluded from RT analyses (percentages do not add up completely since some trials were excluded for more than one reason). Figure 2 displays mean RT as a function of task-order transition (fixed vs. repetition vs. switch), task order (manual-oculomotor vs. oculomotor-manual), and component task (oculomotor vs. manual). RTs, ERs, and response reversal rates are referred to in Table 2.

### Task-order coordination costs

In this set of analyses, we compared performance in fixed-order blocks with performance in order-repetition trials of mixed-order blocks. This was done to assess the general impact of task-order coordination in a setting in which task order is not determined by the order of sequentially presented stimuli but has to be derived from abstract transition (“switch” vs. “repeat”) cues.

*RTs*. Responses were faster in fixed-order blocks (*M* = 583 ms) than in order-repetition trials of mixed-order blocks (*M* = 618 ms), $F\left(1,39\right)=15.59$, $p<.001$, ${\widehat{\eta }}_{p}^{2}=0.286$. These task-order coordination costs were not affected by the specific task order as the two-way interaction between task-order transition and task order was non-significant, $F\left(1,39\right)=0.07$, $p=.795$, ${\widehat{\eta }}_{p}^{2}=0.002$. However, task-order coordination costs were greater in the oculomotor task ($\varDelta M$ = 39 ms, *p* <.001) than in the manual task ($\varDelta M$ = 29 ms, *p* =.003), as indicated by the significant two-way interaction between task-order transition and task, $F\left(1,39\right)=5.62$, $p=.023$, ${\widehat{\eta }}_{p}^{2}=0.126$. Overall, the oculomotor task *M* = 534 ms) was executed faster than the manual task (*M* = 667 ms), $F\left(1,39\right)=174.08$, $p<.001$, ${\widehat{\eta }}_{p}^{2}=0.817$. The significant interaction between task order and task confirmed that the tasks were executed in the correct order, $F\left(1,39\right)=508.97$, $p<.001$, ${\widehat{\eta }}_{p}^{2}=0.929$. Neither the main effect of task order, nor the interaction between task-order transition and task order, nor the three-way interaction were significant (*F*s < 1.78, *p*s > 0.190, ${\widehat{\eta }}_{p}^{2}$s < 0.044).

*Errors*. The main effect of task order transition was non-significant, $F\left(1,39\right)=0.99$, $p=.326$, ${\widehat{\eta }}_{p}^{2}=0.025$. Errors were more frequent in the manual-oculomotor task order (*M* = 4.40%) than in the oculomotor-manual task order (*M* = 3.45%), $F\left(1,39\right)=6.59$, $p=.014$, ${\widehat{\eta }}_{p}^{2}=0.145$. Errors were also more frequent in the oculomotor task (*M* = 6.25%) than in the manual task (*M* = 1.60%), $F\left(1,39\right)=43.37$, $p<.001$, ${\widehat{\eta }}_{p}^{2}=0.527$. However, the two-way interaction between task order and task was significant, $F\left(1,39\right)=23.17$, $p<.001$, ${\widehat{\eta }}_{p}^{2}=0.373$. The difference in errors between the oculomotor and the manual task was smaller in the oculomotor-manual task order ($\varDelta M$ = 3.06%, *p* <.001) than in the manual-oculomotor task order ($\varDelta M$ = 6.24%, *p* <.001) due to less frequent oculomotor errors with the preferred (oculomotor-manual) versus the non-preferred (manual-oculomotor) task order. No other interaction effect was significant (*F*s < 1.03, *p*s > 0.319, ${\widehat{\eta }}_{p}^{2}$s < 0.026).

*Task-order reversals*. Task-order reversals were more frequent in order-repetition trials of mixed-order blocks (8.98%) than in fixed-order blocks (5.49%), indicating task-order coordination costs, $F\left(1,39\right)=30.02$, $p<.001$, ${\widehat{\eta }}_{p}^{2}=0.435$. Neither the main effect of task order, nor the interaction between task-order transition and task order were significant (*F*s < 1.20, *p*s > 0.281, ${\widehat{\eta }}_{p}^{2}$s < 0.031).

### Task-order switch costs

In this set of analyses, we compared order-repetition trials in mixed-order blocks with order-switch trials in mixed-order blocks to assess the nature of task-order representations. Asymmetric costs of a switch to the preferred task order (oculomotor-manual) vs. the non-preferred task-order (manual-oculomotor) would be indicative of task-order representations that are integrated with component task representations. Absence of such asymmetries would indicate a generic representation of task order.

*RTs*. Responses were faster in order-repetition trials (*M* = 618 ms) than in order-switch trials (*M* = 640 ms), $F\left(1,39\right)=12.16$, $p=.001$, ${\widehat{\eta }}_{p}^{2}=0.238$. While the non-significant two-way interaction between task-order transition and task order (*F* < 1, ${\text{B}\text{F}}_{\text{01}}=11.61$) suggests symmetrical costs for switches to either task order, we did find a significant three-way interaction between task-order transition, task order, and task, $F\left(1,39\right)=27.66$, $p<.001$, ${\widehat{\eta }}_{p}^{2}=0.415$. A simple effects analysis revealed that the simple two-way interaction between task-order transition and task order was marginally significant in the oculomotor task ($F\left(1,39\right)=3.74$, $p=.060$, ${\text{B}\text{F}}_{\text{01}}=4.44$), and significant in the manual task ($F\left(1,39\right)=4.44$, $p=.042$, ${\text{B}\text{F}}_{\text{01}}=4.86$), however these interaction went in opposite directions. In the oculomotor-manual task order, only the manual (i.e., second) task showed significant task-order switch costs ($\varDelta M$ = 35 ms, *p* <.001), while the oculomotor (i.e., first) task did not ($\varDelta M$ = 9 ms, *p* =.198). In the manual-oculomotor task order, only the saccade (i.e., second) task showed significant task-order switch costs ($\varDelta M$ = 34 ms, *p* =.008) while the manual (i.e., first) task did not ($\varDelta M$ = 12 ms, *p* =.192). Again, the oculomotor task (*M* = 564ms) was executed faster than the manual task (*M *= 694ms), $F\left(1,39\right)=159.80$, $p<.001$, ${\widehat{\eta }}_{p}^{2}=0.804$. The significant two-way interaction between task order and task confirmed that the tasks were executed in the correct order, $F\left(1,39\right)=522.19$, $p<.001$, ${\widehat{\eta }}_{p}^{2}=0.931$. Neither the main effect of task order, nor the two-way interaction between task-order transition and task were significant (*F*s < 1.93, *p*s > 0.172, ${\widehat{\eta }}_{p}^{2}$s < 0.048).

*Errors*. The main effect of task-order transition was non-significant, $F\left(1,39\right)=1.87$, $p=.179$, ${\widehat{\eta }}_{p}^{2}=0.046$. Errors were more frequent with the manual-oculomotor task order (*M* = 4.13%), than with the oculomotor-manual task order (*M* = 3.11%), $F\left(1,39\right)=6.74$, $p=.013$, ${\widehat{\eta }}_{p}^{2}=0.147$. The two-way interaction between task-order transition and task order was non-significant, indicating an absence of order switch cost asymmetries, $F\left(1,39\right)=0.06$, $p=.811$, ${\widehat{\eta }}_{p}^{2}=0.001$, ${\text{B}\text{F}}_{\text{01}}=8.88$. Errors were more frequent in the oculomotor task (*M* = 5.67%), than in the manual task (*M* = 1.56%), $F\left(1,39\right)=23.72$, $p<.001$, ${\widehat{\eta }}_{p}^{2}=0.378$. The two-way interaction between task order and task was significant, $F\left(1,39\right)=12.40$, $p<.001$, ${\widehat{\eta }}_{p}^{2}=0.241$. The difference in errors between the oculomotor and the manual task was smaller in the oculomotor-manual task order ($\varDelta M$ = 2.84%, *p* <.001) than in the manual-oculomotor task order ($\varDelta M$ = 5.38%, *p* <.001) due to less oculomotor errors with the preferred (oculomotor-manual) versus non-preferred (manual-oculomotor) task order. Neither the two-way interaction between task-order transition and task nor the three-way interaction were significant (*F*s < 1.06, *p*s > 0.311, ${\widehat{\eta }}_{p}^{2}$s < 0.027).

*Task-order reversals*. Task-order reversals were more frequent in order-switch trials (*M* = 11.45%) than in order-repetition trials (*M* = 8.98%), indicating task-order switch costs, $F\left(1,39\right)=15.16$, $p<.001$, ${\widehat{\eta }}_{p}^{2}=0.280$. Task-order reversals were more frequent when the manual-oculomotor task order was required (*M* = 11.39%) than when the oculomotor-manual task order was required (*M* = 9.04%), indicating that the oculomotor-manual task-order was preferred, $F\left(1,39\right)=4.18$, $p=.048$, ${\widehat{\eta }}_{p}^{2}=0.097$. The interaction between task-order transition and task order was non-significant, indicating an absence of order switch-cost asymmetries, $F\left(1,39\right)=1.57$, $p=.218$, ${\widehat{\eta }}_{p}^{2}=0.039$, ${\text{B}\text{F}}_{\text{01}}=5.27$.

## Discussion

The comparisons of performance in fixed-order blocks with that in order-repetition trials of mixed-order blocks consistently showed costs of task-order coordination in RTs and response reversals in both component tasks, replicating typical findings from other studies on task-order control with sequentially presented stimuli (Kübler et al., 2022b; Luria & Meiran, 2003). As was the case in a previous study on the nature of task-order representations in situations with sequentially presented stimuli (Huestegge et al., 2021), these costs did not differ between transitions made to the preferred (vs. non-preferred) task order. These task-order coordination costs can thus be explained in terms of more global executive control processes employed in situations involving frequent switches of response order (vs. constant response order) and are therefore distinct from the more local control processes of switching (vs. repeating) task order in one particular trial (these were previously shown to differ depending on the specific task-order transition made).

When comparing order-repetition trials with order-switch trials in mixed-order blocks (i.e., task-order switch costs) in the current paradigm, we found no evidence for asymmetries in task-order switch costs between the preferred task order and the non-preferred task order but rather substantial evidence (BF_01_ > 5) for the absence of the diagnostic interaction between task-order transition and task order averaged across both component tasks. Further breaking down the significant three-way interaction in the RTs revealed a tendency toward order switch-cost asymmetries in opposite directions for both component tasks. This reflects that switches to either task order incurred significant costs only to the second component task (i.e., the manual task in the oculomotor-manual task order and the oculomotor task in the manual-oculomotor task order). These costs were of a similar size (one millisecond difference). These results thus differ in two notable ways from previous studies. First, the finding of significant task-order switch costs *only* for the second component task was unexpected given that in typical task-order control studies using sequential task stimuli *both* component tasks are usually affected by a task-order switch (Luria & Meiran, 2003, 2006; Strobach et al., 2021) and potential reasons will be discussed in the General Discussion. Second, and more importantly, the previous study by Huestegge et al. (2021) on representations of differentially preferred task orders with sequential stimuli found significant order switch cost asymmetries that went in the *same* direction for both component tasks (i.e., order switch costs were smaller for switches to the oculomotor-manual vs. the manual-oculomotor task order). This pattern was taken as indication for task-order representations that are integrated with component task information (e.g., “oculomotor-manual” vs. “manual-oculomotor”). The present results suggest that in a situation requiring purely top-down task-order control by the means of order transition cues, task order is represented in a more generic manner (e.g., “0–1” vs. “1 − 0”) instead.

Importantly, first, one might object that a generic way of representing task order might have stemmed from the arguably abstract transition cues used in Experiment 1 (i.e., “switch” vs. “repeat”), potentially enticing (or forcing) participants to not incorporate component task information in their task-order set. Second, switches to either task order incurred significant costs only to the second component task, while the first component task was unaffected by these switches. To address these issues, we conducted a second experiment employing task-order cues containing explicit information about task orders (i.e., “gaze-key” vs. “key-gaze”). Notably, any change in the pattern of task-order switch costs would further indicate a strong contextual flexibility regarding the mental representation of task order even within highly comparable settings.

**Table 2:** Descriptive summary statistics of experiment 1 and 2

Experiment	Task order	Task	Mean RT (SEM)			% Errors (SEM)			% Reversals (SEM)		
			Fixed	Repetition	Switch	Fixed	Repetition	Switch	Fixed	Repetition	Switch
Abstract cues	Oculomotor-manual	Oculomotor	384 (11)	420 (14)	428 (14)	5.17 (0.68)	4.79 (0.76)	4.27 (0.59)	5.12 (0.66)	8.14 (0.86)	9.94 (0.79)
		Manual	801 (26)	831 (29)	866 (30)	2.05 (0.38)	1.80 (0.50)	1.57 (0.50)			
	Manual-oculomotor	Oculomotor	644 (24)	687 (26)	721 (26)	7.92 (0.88)	7.11 (0.99)	6.53 (1.02)	5.85 (0.96)	9.82 (1.14)	12.96 (1.33)
		Manual	505 (20)	534 (22)	546 (22)	1.14 (0.21)	1.41 (0.42)	1.47 (0.58)			
Explicit cues	Oculomotor-manual	Oculomotor	388 (11)	465 (21)	481 (22)	5.97 (0.79)	5.85 (0.74)	5.39 (0.68)	6.18 (0.74)	8.53 (1.20)	12.59 (1.48)
		Manual	810 (32)	877 (42)	904 (42)	2.36 (0.31)	1.82 (0.28)	1.66 (0.30)			
	Manual-oculomotor	Oculomotor	630 (24)	715 (34)	761 (35)	9.12 (0.81)	7.68 (0.79)	6.15 (0.80)	5.63 (0.76)	11.29 (1.82)	17.54 (2.05)
		Manual	481 (15)	557 (24)	589 (26)	2.21 (0.59)	1.10 (0.24)	1.13 (0.25)			

*Note* Fixed = Fixed task-order blocks, Repetition = Task-order repetition trials in mixed task-order blocks, Switch = Task-order switch trials in mixed task-order blocks. Reversal rates are identical for both component tasks for a given task order. Standard errors of the mean (SEM) are presented in parentheses

## Experiment 2

In Experiment 2, we employed explicit task-order cues to gauge whether the result pattern in Experiment 1 was caused by the abstract transition cues used in this first experiment.

### Methods

Forty new participants (mean age = 24.60 years, SD = 5.10, 60% female) were recruited. Four data sets were replaced according to the same criteria as in Experiment 1.

Materials and procedure were identical to Experiment 1, except for the fact that we now used written (white Times New Roman font, size 20) explicit cues to signal the required task order in every trial (see Fig. 1C). The oculomotor-manual task order was cued by “Blick - Taste” (meaning “Gaze - Key”) and the manual-oculomotor task order was cued by “Taste - Blick” (meaning “Key - Gaze”).

We analyzed the data in the same way as in Experiment 1 with two sets of analyses (task-order coordination costs and task-order switch costs) for each of the three dependent variables (RTs, errors, order reversals). From the RT analyses, 29.44% of all trials were excluded (2.08% first trials of each block, 27.37% error trials and trials following an error, 2.33% outliers). Figure 3 displays mean RT as a function of task-order transition (fixed vs. repetition vs. switch), task order (manual-oculomotor vs. oculomotor-manual), and component task (oculomotor vs. manual).

### Results

**Fig. 3:**
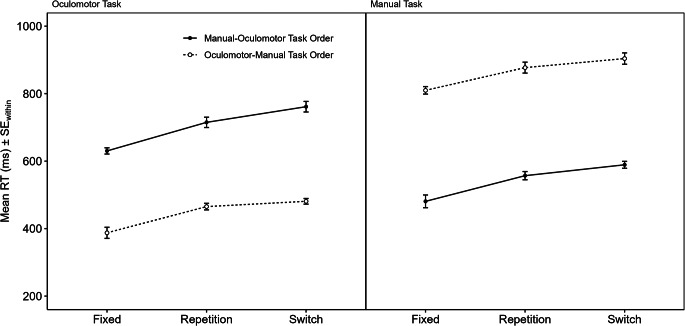
Reaction times (RT) of Experiment 2 as a function of Task-Order Transition (Fixed vs. Repetition vs. Switch), Task Order (Manual-Oculomotor vs. Oculomotor-Manual), and Task (Oculomotor vs. Manual). *Note* For task-order switch cost asymmetries indicative of integrated task-order representations, the differences between Switch and Repetition trials would need to be greater for the Manual-Oculomotor Task Order (solid black lines) than for the Oculomotor Manual Task Order (broken lines) in *both* Tasks. Error bars represent within-subject standard errors following Cousineau-Morey corrections (O’Brien & Cousineau, 2014)

### Task-order coordination cost

Again, to assess the general impact of task-order coordination with explicitly cued task order, we compared performance in fixed-order blocks with performance in order-repetition trials in mixed-order blocks.

*RTs*. Responses were faster in fixed-order blocks (*M* = 577 ms) than in order-repetition trials of mixed-order blocks (*M* = 654 ms), $F\left(1,39\right)=34.84$, $p<.001$, ${\widehat{\eta }}_{p}^{2}=0.472$. These task-order coordination costs were not affected by the specific task order as the two-way interaction between task-order transition and task order was non-significant, $F\left(1,39\right)=0.43$, $p=.515$, ${\widehat{\eta }}_{p}^{2}=0.011$. However, task-order coordination costs were slightly greater in the oculomotor task ($\varDelta M$ = 81 ms, *p* <.001) than in the manual task ($\varDelta M$ = 72 ms, *p* =.003), as indicated by the marginally significant two-way interaction between task-order transition and task, $F\left(1,39\right)=3.94$, $p=.054$, ${\widehat{\eta }}_{p}^{2}=0.092$. Overall, the manual-oculomotor task order (*M* = 596ms) was completed faster than the oculomotor-manual task order (*M* = 635ms), $F\left(1,39\right)=13.88$, $p<.001$, ${\widehat{\eta }}_{p}^{2}=0.263$ and the oculomotor task (*M* = 550ms) was executed faster than the manual task (*M* = 681ms), $F\left(1,39\right)=232.87$, $p<.001$, ${\widehat{\eta }}_{p}^{2}=0.857$. The significant two-way interaction between task order and task confirmed that the tasks were executed in the correct order, $F\left(1,39\right)=279.36$, $p<.001$, ${\widehat{\eta }}_{p}^{2}=0.877$. Neither the two-way interaction between task-order transition and task order, nor the three-way interaction were significant (*F*s < 1, *p*s > 0.514, ${\widehat{\eta }}_{p}^{2}$s < 0.012).

*Errors*. Errors were more frequent in fixed-order blocks (*M* = 4.92%) than in order-repetition trials of mixed-order blocks (*M* = 4.11%), $F\left(1,39\right)=5.72$, $p=.022$, ${\widehat{\eta }}_{p}^{2}=0.128$, indicating a task-order coordination *benefit*. However, the marginally significant two-way interaction between task-order transition and task-order indicated that this benefit was significant only in the manual-oculomotor task order ($\varDelta M$ = -1.28%, *p* =.012) but not in the oculomotor-manual task order ($\varDelta M$ = -0.33%, *p* =.353), $F\left(1,39\right)=3.47$, $p=.070$, ${\widehat{\eta }}_{p}^{2}=0.082$. Errors were also more frequent in the manual-oculomotor task order (*M* = 5.03%) than in the oculomotor-manual task order (*M* = 4.00%), $F\left(1,39\right)=15.20$, $p<.001$, ${\widehat{\eta }}_{p}^{2}=0.280$. Errors were more frequent in the oculomotor task (7.16%) than in the manual task (1.87%), $F\left(1,39\right)=75.53$, $p<.001$, ${\widehat{\eta }}_{p}^{2}=0.659$. The two-way interaction between task order and task was significant, $F\left(1,39\right)=14.09$, $p<.001$, ${\widehat{\eta }}_{p}^{2}=0.265$. The difference in errors between the oculomotor and the manual task was smaller in the oculomotor-manual task order ($\varDelta M$ = 3.82%, *p* <.001) than in the manual-oculomotor task order ($\varDelta M$ = 6.75%, *p* <.001) due to less oculomotor errors with the preferred (oculomotor-manual) versus the non-preferred (manual-oculomotor) task order. Neither the two-way interaction between task-order transition and task nor the three-way interaction were significant (*F*s < 1, *p*s > 0.359, ${\widehat{\eta }}_{p}^{2}$s < 0.022).

*Task-order reversals*. Task-order reversals were more frequent in order-repetition trials of mixed-order blocks (9.91%) than in fixed-order blocks (5.90%), indicating task-order coordination costs, $F\left(1,39\right)=17.66$, $p<.001$, ${\widehat{\eta }}_{p}^{2}=0.312$. The marginally significant interaction between task-order transition and task indicated that task-order coordination costs were slightly greater in the manual-oculomotor task order ($\varDelta M$ = 5.66%, *p* =.001) than in the oculomotor-manual task order ($\varDelta M$ = 2.36%, *p* =.003), $F\left(1,39\right)=3.97$, $p=.053$, ${\widehat{\eta }}_{p}^{2}=0.092$. The main effect of task order was non-significant, $F\left(1,39\right)=0.74$, $p=.394$, ${\widehat{\eta }}_{p}^{2}=0.019$.

### Task-order switch costs

Order-repetition trials in mixed-order blocks were compared to order-switch trials in mixed-order blocks. The presence or absence of asymmetries in task-order switch costs for the preferred (i.e., oculomotor-manual) versus the non-preferred (i.e., manual-oculomotor) task orders were again used to infer the nature of task-order representations in a situation requiring task-order coordination and in which task order is determined by an *explicit* task order cue.

*RTs*. Responses were faster in order-repetition trials (*M* = 654 ms) than in order-switch trials (*M* = 684 ms), $F\left(1,39\right)=30.34$, $p<.001$, ${\widehat{\eta }}_{p}^{2}=0.438$. We found no significant two-way interaction between task-order transition and task order (*F* < 1.97, ${\text{B}\text{F}}_{\text{01}}=6.74$), suggesting an absence of order switch-cost asymmetries. However, there was a significant three-way interaction between task-order transition, task order, and task, $F\left(1,39\right)=6.22$, $p=.017$, ${\widehat{\eta }}_{p}^{2}=0.137$. A simple effects analysis revealed that the simple two-way interaction between task-order transition and task order was marginally significant in the oculomotor task ($F\left(1,39\right)=4.04$, $p=.051$, ${\text{B}\text{F}}_{\text{01}}=3.77$), and non-significant in the manual task ($F\left(1,39\right)=0.19$, $p=.663$, ${\text{B}\text{F}}_{\text{01}}=7.89$). In contrast to Experiment 1, in the oculomotor-manual task order, both the oculomotor (i.e., first) task ($\varDelta M$ = 16 ms, *p* =.008) and the manual (i.e., second) task ($\varDelta M$ = 27 ms, *p* <.001) showed significant task-order switch costs. In the manual-oculomotor task order, both the manual (i.e., first) task ($\varDelta M$ = 32 ms, *p* <.001) and the oculomotor (i.e., second) task ($\varDelta M$ = 46 ms, *p* =.001) showed significant task-order switch costs. Note that despite the absence of significant interaction effects, task-order switch costs were numerically smaller when switching to the preferred (oculomotor-manual) task order than when switching to the non-preferred (manual-oculomotor) task order. Again, the oculomotor task (*M* = 606 ms) was, overall, executed faster than the manual task (*M* = 732 ms), $F\left(1,39\right)=198.29$, $p<.001$, ${\widehat{\eta }}_{p}^{2}=0.836$. The significant two-way interaction between task order and task confirmed that tasks were executed in the correct order, $F\left(1,39\right)=273.22$, $p<.001$, ${\widehat{\eta }}_{p}^{2}=0.875$. Overall, the manual-oculomotor task-order (*M* = 656 ms) was completed faster than the oculomotor-manual task order (*M* = 682 ms), $F\left(1,39\right)=5.39$, $p=.026$, ${\widehat{\eta }}_{p}^{2}=0.121$. The two-way interaction between task-order transition and task was non-significant, $F\left(1,39\right)=0.10$, $p=.751$, ${\widehat{\eta }}_{p}^{2}=0.003$.

*Errors*. Errors were more frequent in order-repetition trials (*M* = 4.11%) than in order-switch trials (*M* = 3.58%), $F\left(1,39\right)=6.20$, $p=.017$, ${\widehat{\eta }}_{p}^{2}=0.137$, indicating a task-order switch *benefit*. The non-significant two-way interaction between task-order transition and task order indicated an absence of asymmetries in this benefit between specific task orders, $F\left(1,39\right)=0.66$, $p=.423$, ${\widehat{\eta }}_{p}^{2}=0.017$, ${\text{B}\text{F}}_{\text{01}}=7.60$. The significant two-way interaction between task-order transition and task indicated that this benefit was significant only in the oculomotor task ($\varDelta M$ = -0.99%, *p* =.013) but not in the manual task ($\varDelta M$ = -0.06%, *p* =.766), $F\left(1,39\right)=4.33$, $p=.044$, ${\widehat{\eta }}_{p}^{2}=0.100$. Errors were more frequent in the oculomotor task (*M* = 6.27%), than in the manual task (*M* = 1.43%), $F\left(1,39\right)=59.18$, $p<.001$, ${\widehat{\eta }}_{p}^{2}=0.603$. The two-way interaction between task order and task was significant. The difference in errors between the oculomotor and the manual task was smaller in the oculomotor-manual task order ($\varDelta M$ = 3.88%, *p* <.001) than in the manual-oculomotor task order ($\varDelta M$ = 5.80%, *p* <.001), $F\left(1,39\right)=11.90$, $p<.001$, ${\widehat{\eta }}_{p}^{2}=0.234$ due to less frequent oculomotor errors with the preferred (oculomotor-manual) versus the non-preferred (manual-oculomotor) task order. Neither the main effect of task order, nor the two-way interaction between task-order transition and task were significant ($Fs<1.57$, $ps>.218$, ${\widehat{\eta }}_{p}^{2}s<0.039$).

*Task-order reversals*. Task-order reversals were more frequent in order-switch trials (*M* = 15.07%) than in order-repetition trials (*M* = 9.91%), indicating task-order switch costs, $F\left(1,39\right)=49.93$, $p<.001$, ${\widehat{\eta }}_{p}^{2}=0.561$. Task-order reversals were slightly more frequent when the manual-oculomotor task order was required (*M* = 14.42%) than when the oculomotor-manual task order was required (*M* = 10.56%), indicating preference for the oculomotor-manual task order, $F\left(1,39\right)=3.97$, $p=.053$, ${\widehat{\eta }}_{p}^{2}=0.092$. The interaction between task-order transition and task order was significant, $F\left(1,39\right)=4.64$, $p=.037$, ${\widehat{\eta }}_{p}^{2}=0.106$. The task-order switch cost in task-order reversals was greater for switches to the manual-oculomotor task order ($\varDelta M$ = 6.26%, *p* <.001) than for switches to the oculomotor-manual task order ($\varDelta M$ = 4.05%, *p* <.001).

## Discussion

Experiment 2 used task-order cues containing explicit information about the to-be-scheduled task order. As in Experiment 1, we found significant global task-order coordination costs when comparing fixed-order blocks to order-repetition trials in mixed-order blocks (Kübler et al., 2022b; Luria & Meiran, 2003). This indicates that the abstract transition cues used in the first experiment were not the main reason for task-order coordination difficulties. Importantly, when comparing order-repetition trials with order-switch trials in mixed-order blocks (i.e., task-order switch costs), we found a markedly different pattern of results compared to Experiment 1. In Experiment 2, task-order switch costs in the RTs were significant in *both* the first and the second component task of each task order, more in line with previous task-order control studies employing sequentially presented stimuli (Luria & Meiran, 2003, 2006; Strobach et al., 2021), suggesting that participants represented task order in a different way than in Experiment 1. Interestingly, both task-order coordination costs and task-order switch costs as well as the overall RT level were greater in Experiment 2 compared to Experiment 1. This indicates that the coordination of explicitly cued task orders was more difficult than when abstract transition order transition cues were used, potentially due to stronger crosstalk between task order representations formed in the former setting. However, in contrast to Experiment 1, we also observed an unexpected task-order switch *benefit* in the rate of oculomotor errors which might indicate a speed-accuracy trade-off. This benefit was only significant in the oculomotor task while both component tasks showed stronger task-order coordination and switch costs compared to the first Experiment. Most importantly, we found a numerical trend toward asymmetries in task-order switch costs in RTs with smaller costs in both component tasks (in contrast, there was no such trend in the second component task of Experiment 1) for switches to the preferred (oculomotor-manual) task order compared to the non-preferred (manual-oculomotor) task order with no counteracting trend in the directional errors. While the three-way interaction (RTs) was clearly significant, indicating that the interaction between task-order transition and task order significantly differed between the oculomotor and manual domains, the *direction* of these interaction was the same for both component tasks. Critically, this pattern of results contrasts with Experiment 1 of the present study but is in line with the study by Huestegge et al. (2021), using sequentially presented stimuli and therefore indicates an integration of component task information and task-order representations. This conclusion should be taken with a grain of salt of course since the simple two-way interaction between task-order transition and task order was only marginally significant in the oculomotor task and non-significant in the manual task with substantial evidence for the absence of an interaction in both cases (BF_01_ > 3). Nevertheless, the descriptive pattern of results was quite consistent, and clearly different from the one obtained in Experiment 1. Potential reasons for the non-significance of task-order switch cost asymmetries are discussed in the General Discussion.

## General discussion

The present study investigated task-order representations in a situation involving unpredictably cued switches between differentially preferred task orders with simultaneously presented task stimuli. This setting did not allow for classic bottom-up effects (of stimulus presentation order) to emerge, but rather called for purely top-down driven task-order control. The results can be summarized as follows. Across experiments, preference for the oculomotor-manual (vs. manual-oculomotor) task order was confirmed by the generally lower order reversal rates in the former (vs. latter) task order (mainly in mixed-order blocks), thereby serving as a manipulation check. Note, however, that this effect was numerically smaller than in previous studies using sequentially presented task stimuli, which could be a reason for obtaining smaller effects of the specific task order on task-order switch costs (cf., Pieczykolan & Huestegge, 2019). Furthermore, we found significant task-order coordination costs and task-order switch costs in both Experiment 1 and Experiment 2, suggesting that with abstract transition cues as well as explicit order cues, distinct task-order representations had to be maintained and coordinated in working memory in mixed-order blocks. Interestingly, however, task-order coordination costs (in RTs) were greater when explicit task-order cues were used (Experiment 2) than when abstract order transition cues were used (Experiment 1). This suggests different degrees of interference between task-order representations formed in these two settings. Most importantly, the pattern of task-order switch costs substantially differed between experiments. In Experiment 1, the significant three-way interaction in RTs indicated task-order switch cost asymmetries for the preferred versus the non-preferred task order that went in opposite directions for both component tasks. That is, we found task-order switch costs only for the secondly executed component task of either task order (i.e., the manual task in the oculomotor-manual task order and the oculomotor task in the manual-oculomotor task order). These costs were comparable in size, suggesting that participants formed a rather generic representation of task order (i.e., switches to either task order were conducted similarly). In Experiment 2, in contrast, we found task-order switch costs in *both* component tasks. Furthermore, there was a tendency toward smaller costs for switches to the preferred versus the non-preferred task order (in particular for the oculomotor task, as indicated by the significant three-way interaction for RTs). This pattern mirrors the results in the Huestegge et al. (2021) study and suggests that task order was represented in a more integrated manner with component task information (i.e., switches to the different task orders were conducted with different ease). A potential reason for the unexpected limitation of switch costs to the second component task in Experiment 1 might be that participants only fully prepared for the first task with the transition cues, and postpone full preparation for the second task. In contrast, the explicit cuing of the task pair in Experiment 2 may have induced participants to prepare the two tasks as a pair, preparing for both tasks in a more parallel fashion. Together, these findings point toward a strong flexibility in mental task-order representations and the way different task orders are coordinated, depending on the exact cuing procedure employed when stimuli are presented simultaneously without providing bottom-up task-order information.

In most studies on task-order control with sequential stimulus presentation, task-order switch costs emerged in both component tasks, even when bottom-up influences are reduced (e.g., Luria & Meiran, 2006, Experiment 2), which is usually taken as evidence for distinct, higher-order representations of task order (Hirsch et al., 2017, 2018). If participants would only represent each task individually, one would not expect any task-order switch costs (or even task-order switch benefits) for the first component task since a task-order switch would be accompanied by a (local) task repetition across trials (e.g., oculomotor-[manual → manual]-oculomotor). In Experiment 1 of the present study, however, significant task-order switch costs (in RTs) were only found for the second task of either task order but not for the first. This, however, cannot be interpreted as indicating that no representations of task-order were formed here, since in situations with an abstract transition cue and simultaneous stimuli, top-down task-order control should rely even *more* on active task-order representations than in situations in which stimulus order can act as bottom-up reference. However, we did not find significant asymmetries of task-order switch costs (in the second task) for transitions to the preferred (oculomotor-manual) vs. non-preferred (manual-oculomotor) task order. This indicates that when using abstract order transition cues, task-order representations did not contain information about component task characteristics such as the differentially dominant effector systems employed, which would render one task order easier to switch to. In contrast, task order was likely represented in a more generic manner containing only generic order information (e.g., “0–1” vs. “1 − 0”) or potentially even only order transition information (e.g., “repeat order” vs. “switch order”), with switches to either task order thereby incurring the same cost. However, it remains for future research to examine why such generic representations of task order led to task-order switch costs only in the second but not the first component tasks as the present data cannot account for this unexpected finding.

Generic task-order representations might have been especially promoted by the transition cues employed in Experiment 1. However, as the results of Experiment 2 showed, top-down controlled task order is not necessarily represented in such a manner. Here, we found significant task-order switch costs in *both* component tasks, as is common in situations with sequentially presented stimuli (e.g., De Jong, 1995; Luria & Meiran, 2003, 2006; Strobach et al., 2021). Moreover, we found a trend toward cost asymmetries between switches to the preferred vs. the non-preferred task order in the same direction as in the earlier study by Huestegge et al. (2021). This suggests that participants have integrated component task information (i.e., the effector systems employed) in their task-order representation when we used explicit order cues to signal task order. Still, even though the pattern is highly consistent with the data of Huestegge and colleagues (2021), the asymmetries in the present study were not as pronounced. It is unlikely that the preference for the oculomotor-manual (vs. manual-oculomotor) task order was not present in the current study given the consistent pattern of reversal rates in mixed-order blocks. However, the effects of a differential preference on task-order switch costs could have been attenuated in the present study since we used rather long time intervals between the cue and the task stimuli to provide participants with sufficient time to process the complex transition cues in Experiment 1. Previous research showed that even with long intervals between a cue and the first of two sequentially presented stimuli, significant task-order switch costs in both component tasks should be observed (Luria & Meiran, 2003). Nevertheless, participants might have used the ample preparation time to resolve much of the conflict between the differentially preferred task orders before executing the first task. This makes it even more astonishing that we still observed a consistent asymmetry (at least descriptively).

These results should be discussed in the context of another recent study that has suggested evidence for generic task-order representations even in a situation with stimulus order as the bottom-up guide for response order (Kübler et al., 2022a). Kübler and colleagues found task-order switch costs for different types of visual/ auditory tasks, regardless of whether the task pair in the current trial (i.e., being transitioned to) contained the *exact* same or a different type of visual/ auditory tasks as in the previous trials (i.e., being transitioned away from). This was interpreted as evidence for rather generic task-order representations that do not contain information about the *exact* component task being scheduled (e.g., visual(A)-auditory → auditory-visual(B) but rather only general order information (e.g., visual-auditory → auditory-visual). However, note that in this study different tasks were defined by the type of visual/ auditory stimulus information instead of differentially dominant responses, thus likely not creating any meaningful preferences for one task (response) order over another. Thereby, potential asymmetries of task-order switch costs might have been masked in this study as they were potentially attenuated in Experiment 2 of our study (cf. discussion of Experiment 1 in Huestegge et al., 2021). Furthermore, in the current study, the notion of *generic* task-order representations obtained in Experiment 1 has a different meaning than in the study by Kübler and colleagues (2022a) in that it refers to completely generic representations of task-order (i.e., “0–1” vs. “1 − 0”, potentially even “repeat order” vs. “switch order”) instead of task-order representations containing “only” no information of the *exact* types of component task being ordered (i.e., in the Kübler et al. study, a representation such as “visual task first” would already count as “generic”, even though the visual nature of the task could be part of the representation). While we do not claim to refute the findings of the Kübler et al. study, in light of the present findings, it seems possible that even in their study, task-order representations contained information about the (types of) component tasks being ordered, if not about their specific stimulus-response mapping rules. Future studies should be dedicated to refining answers to the question of what actually constitutes an “generic” vs. an “integrated” representation. Nevertheless, the present study indicates that even in two highly comparable situations containing differentially preferred task orders and the need to control task-order in a purely top-down manner, task-order representations appear to be quite flexible, as mental representations are potentially in general (Raettig & Huestegge, 2021).

## Conclusion

To conclude, the present study for the first time demonstrated the effects of task-order control requirements in the absence of stimulus order as a bottom-up guide to response-order control. We replicated findings of impaired performance in situations calling for frequent (top-down controlled) task-order switches compared with situations requiring a constant task order (i.e., task-order coordination costs). Importantly, we showed that in such a situation without any bottom-up source of information (stimulus order) to determine task scheduling, task order can be represented in a generic fashion or integrated with features of the specific component tasks depending on the particular cuing procedure employed. These findings highlight an astonishing flexibility of mental task-order representations and task-order control that appear to be tailored to the specific environmental (situational) demands.

## Data Availability

The datasets used and/or analysed during the current study are publicly available at https://osf.io/mwu8r/.
